# Supplementary figures and images for: A hybrid de novo genome assembly of the honeybee, Apis mellifera, with chromosome-length scaffolds
Source: BMC Genomics. 2019 Apr 8;20:275. doi: 10.1186/s12864-019-5642-0 (PMC6454739; doi:10.1186/s12864-019-5642-0)

A

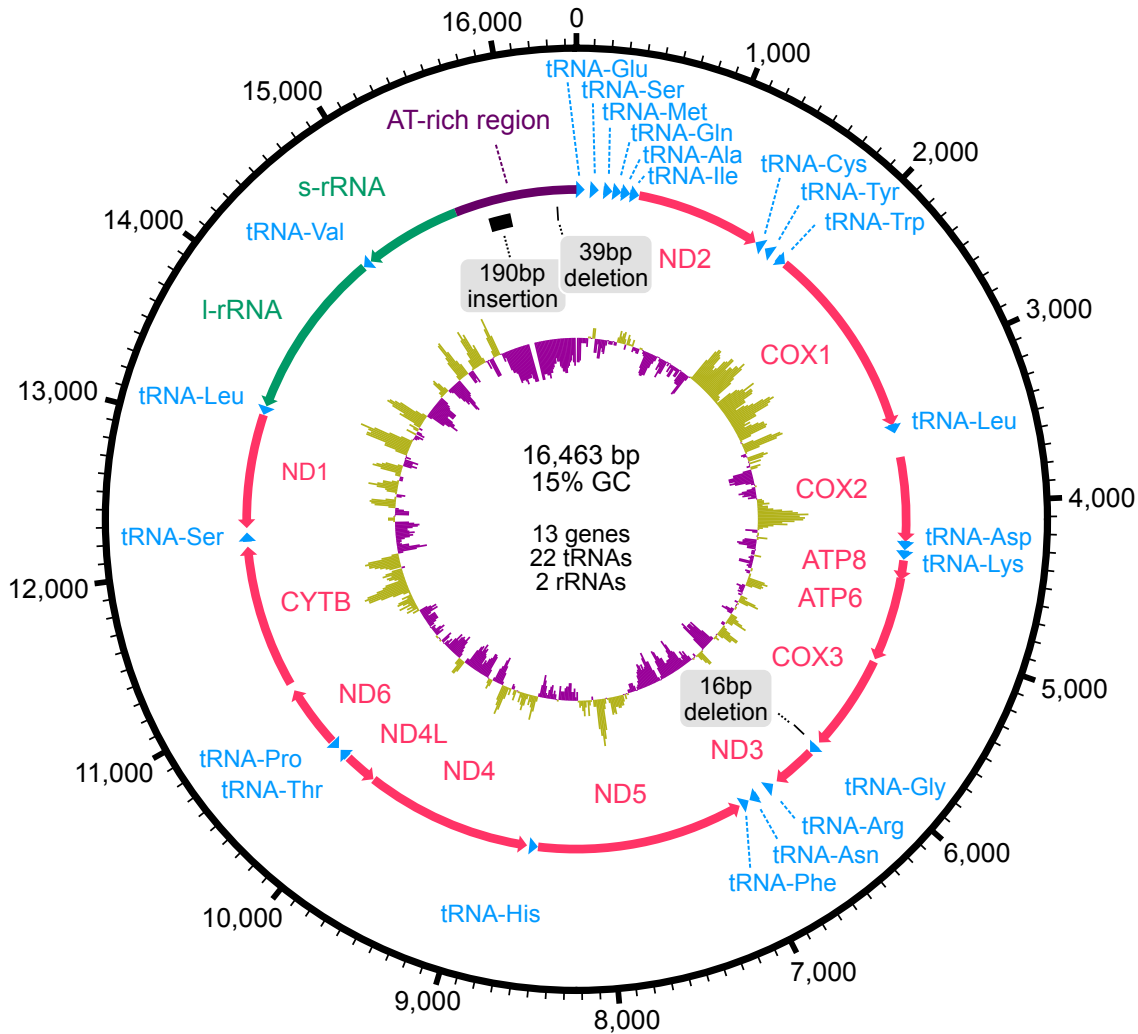

B

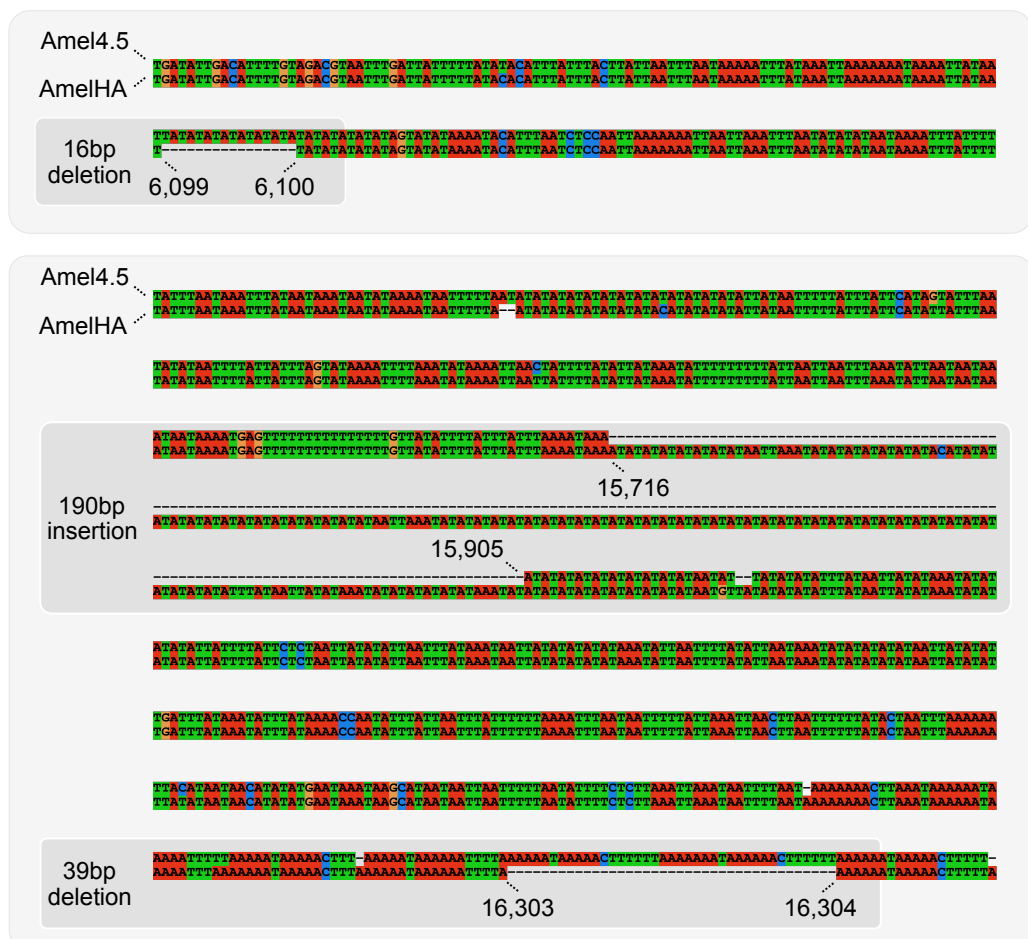

Supplement: Supplementary file 2 — Figure S2. A map of the mitochondrial sequence in the hybrid assembly (Amel_HAv3). A) Summary statistics are presented in the center of the circularized sequence, followed by a 100 bp sliding-window (20 bp steps) bar-plot of GC-content relative to the mitochondrial average (15%). Major structural indels between Amel_HAv3 and Amel_4.5 mitochondrial sequences are indicated as black boxes. The order and orientation of the coding genes (pink), rRNAs (green), tRNAs (blue) are illustrated as arrows. The AT-rich region is indicated in deep purple. Coordinates are given in the outer circle. B) Alignments between Amel_HAv3 and Amel_4.5 illustrate base-level coordinates and composition of the structural variants highlighted in A. (PDF 441 kb) [file 12864_2019_5642_MOESM2_ESM.pdf]

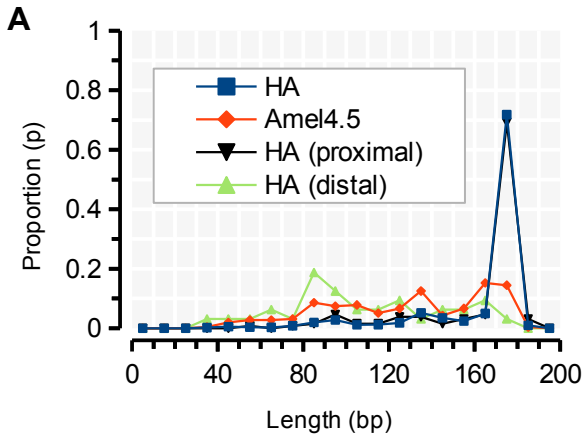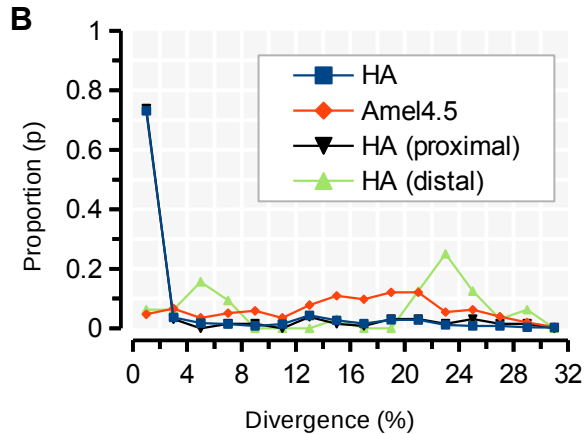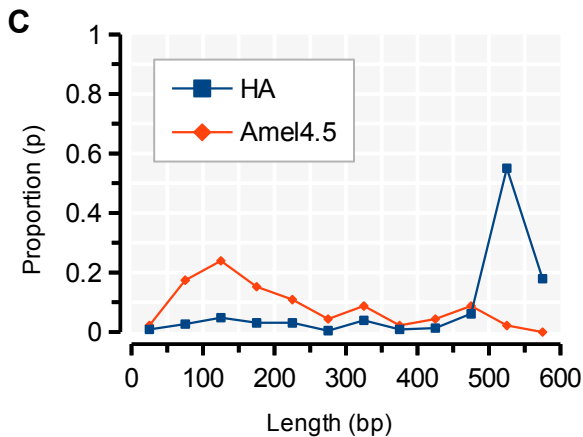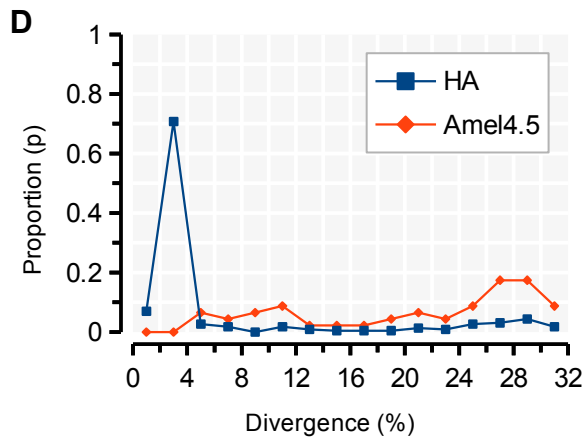

Supplement: Supplementary file 3 — Figure S3. Properties of AluI (176 bp) and AvaI (547 bp) RepeatMasker matches in the hybrid assembly (Amel_HAv3) and Amel_4.5. A) The length distribution of masked AluI repeats in either assembly. These are further subdivided according proximal or distal ends of chromosomes in Amel_HAv3. B) The distribution of sequence divergence from the canonical AluI motif. Classes and colors as in A. C) The length distribution of AvaI matches. D) The distribution of sequence divergence from the canonical AvaI motif. Classes and colors as in C. (PDF 40 kb) [file 12864_2019_5642_MOESM3_ESM.pdf]

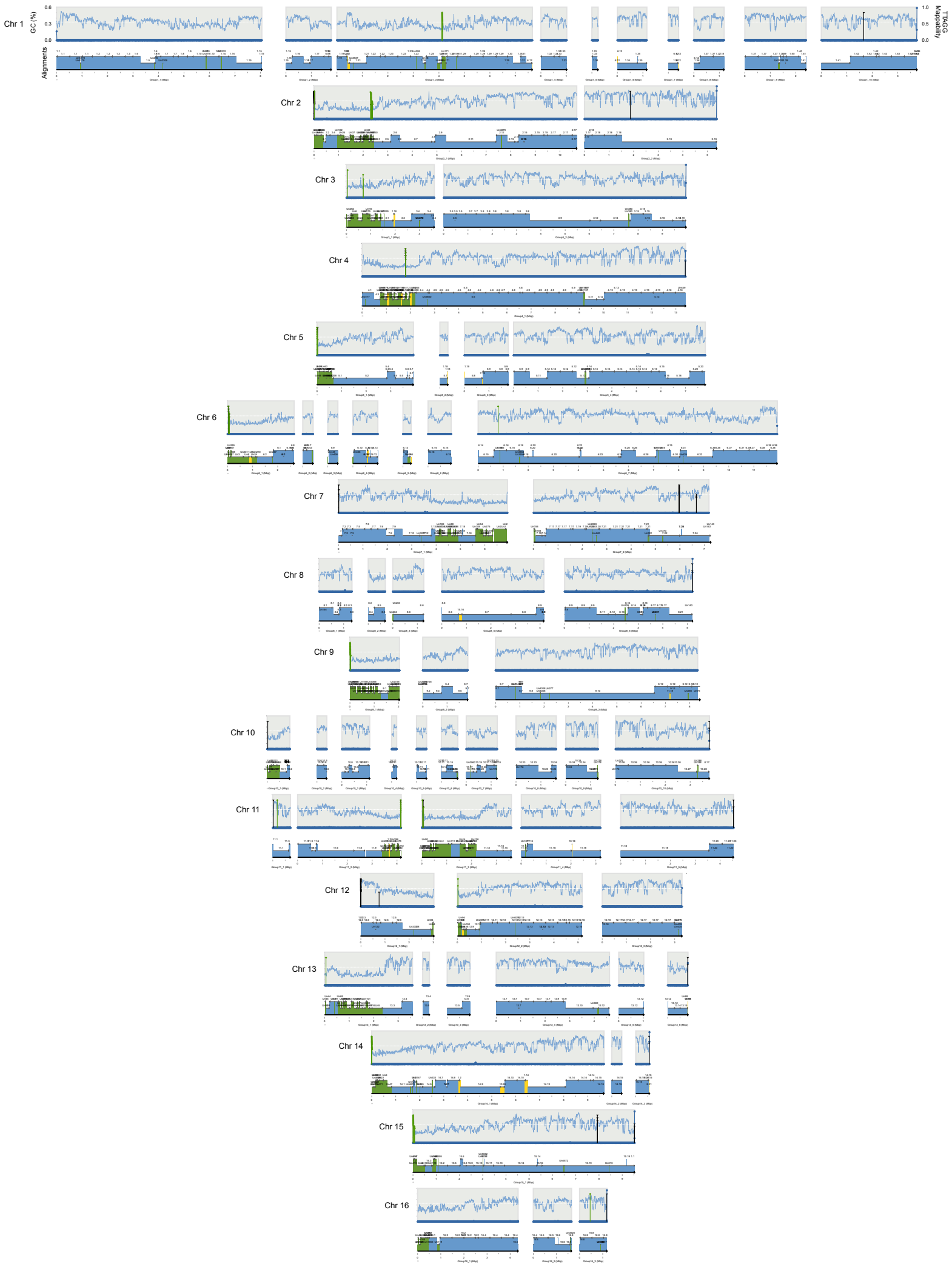

Supplement: Supplementary file 5 — Figure S4. Genome-wide Satsuma alignments between hybrid assembly (Amel_HAv3) and Amel_4.5. A) Alignments across every chromosome. Upper plot: genome-wide GC-content is indicated with a white dashed line and local %GC is mapped across all chromosomes (10kbp non-overlapping windows; light-blue curve on y1-axis). The density of telomeric TTAGG repeats is shown on the y2-axis (10kbp non-overlapping windows; dark-blue curve with circles). Average GEM mappability scores is show on y2-axis (10kbp non-overlapping windows; grey curve). Lower plot: Amel_4.5 scaffolds (upper grey arrows) aligned against Amel_HAv3 contigs (lower black arrows). Coordinates are Mbp-scale. Colors indicate aligned blocks (blue = alignments between sequences that occur on the same chromosome in both assemblies; green = alignments between sequences that are anchored to chromosomes in Amel_HAv3 but were unplaced in Amel_4.5; yellow = alignments between sequences that have switched chromosomes). White spaces are unaligned regions. The locations of centromeric AvaI (green) and telomeric AluI (black) clusters, respectively, are marked along chromosomes. B) As in A, but for unplaced fragments. (ZIP 2983 kb) [file 12864_2019_5642_MOESM5_ESM.zip › Supp_Fig_4A.Synteny_along_chromosomes.pdf]

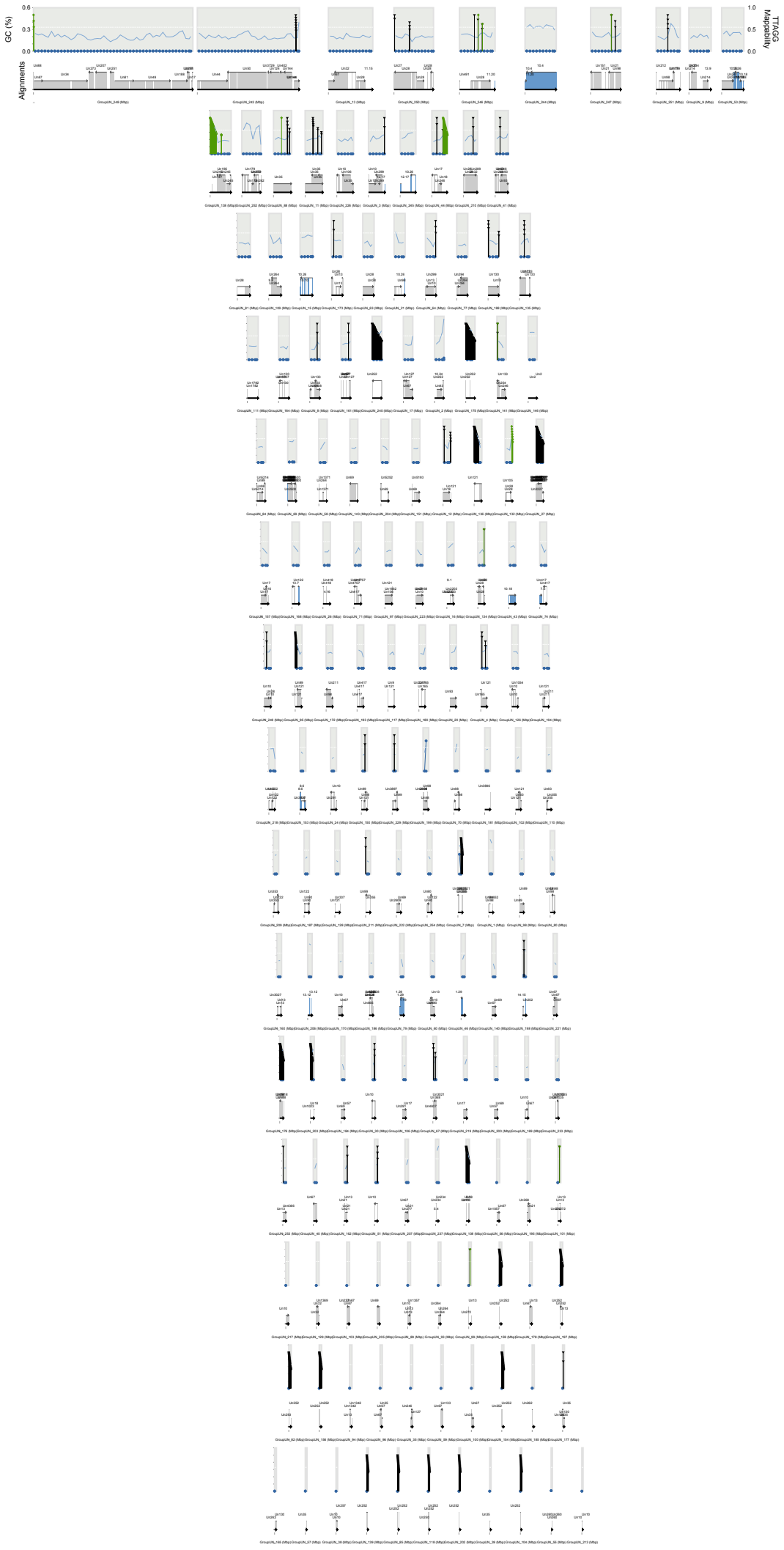

Supplement: Supplementary file 5 — Figure S4. Genome-wide Satsuma alignments between hybrid assembly (Amel_HAv3) and Amel_4.5. A) Alignments across every chromosome. Upper plot: genome-wide GC-content is indicated with a white dashed line and local %GC is mapped across all chromosomes (10kbp non-overlapping windows; light-blue curve on y1-axis). The density of telomeric TTAGG repeats is shown on the y2-axis (10kbp non-overlapping windows; dark-blue curve with circles). Average GEM mappability scores is show on y2-axis (10kbp non-overlapping windows; grey curve). Lower plot: Amel_4.5 scaffolds (upper grey arrows) aligned against Amel_HAv3 contigs (lower black arrows). Coordinates are Mbp-scale. Colors indicate aligned blocks (blue = alignments between sequences that occur on the same chromosome in both assemblies; green = alignments between sequences that are anchored to chromosomes in Amel_HAv3 but were unplaced in Amel_4.5; yellow = alignments between sequences that have switched chromosomes). White spaces are unaligned regions. The locations of centromeric AvaI (green) and telomeric AluI (black) clusters, respectively, are marked along chromosomes. B) As in A, but for unplaced fragments. (ZIP 2983 kb) [file 12864_2019_5642_MOESM5_ESM.zip › Supp_Fig_4B.Synteny_between_unplaced_contigs.pdf]

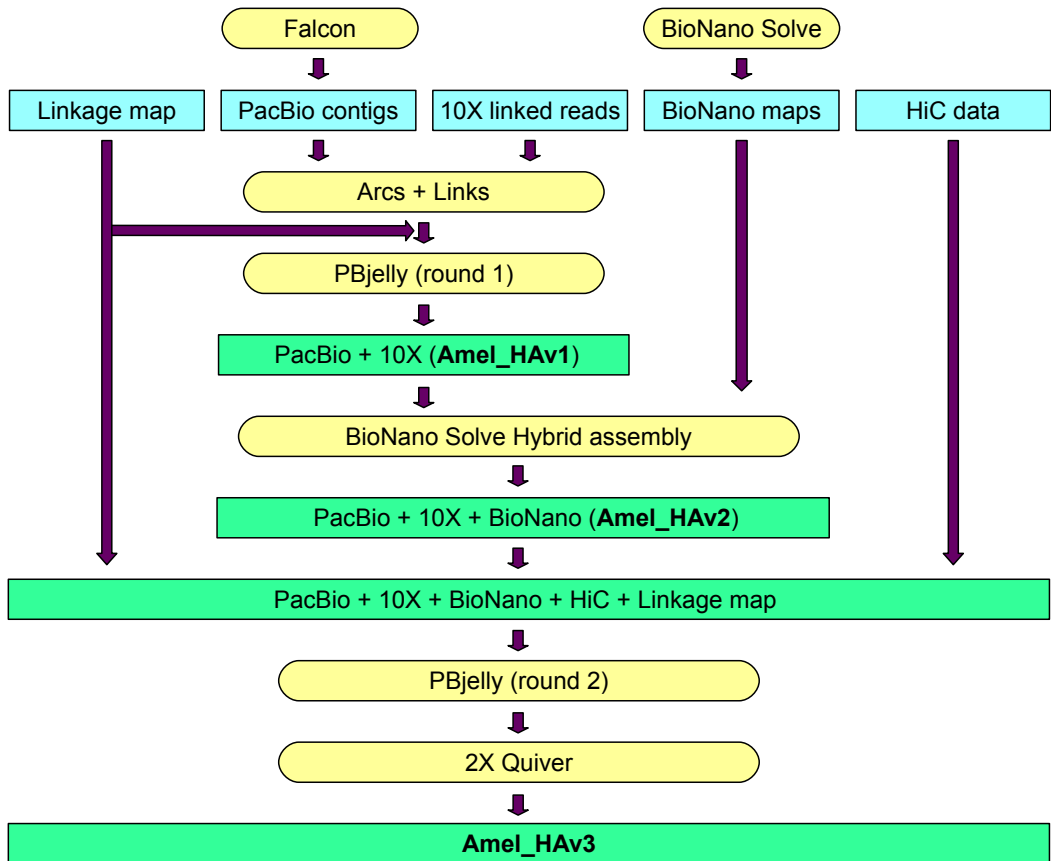

Supplement: Supplementary file 6 — Figure S1. Assembly pipeline. Flowchart illustrating the assembly process. Data sources used as input are displayed in cyan, methods are displayed in yellow, and assembly versions are displayed in green. The final assembly, version 3, is designated Amel_HAv3 (PDF 29 kb) [file 12864_2019_5642_MOESM6_ESM.pdf]
