# Supplementary material for: A hybrid de novo genome assembly of the honeybee, Apis mellifera, with chromosome-length scaffolds
Source: BMC Genomics. 2019 Apr 8;20:275. doi: 10.1186/s12864-019-5642-0 (PMC6454739; doi:10.1186/s12864-019-5642-0)
Supplement: Supplementary file 4 — Figure S5. Density of repeat elements in different genomic regions in the hybrid assembly (Amel_HAv3). Density of interspersed and tandem repeats in different Amel_HAv3 regions, with or without matching sequence in Amel_4.5 (see Fig. 5A for detailed definitions). 95% confidence intervals were generated from bootstrapping randomly extracted blocks of 1 kbp. (PDF 31 kb) [file 12864_2019_5642_MOESM4_ESM.pdf]

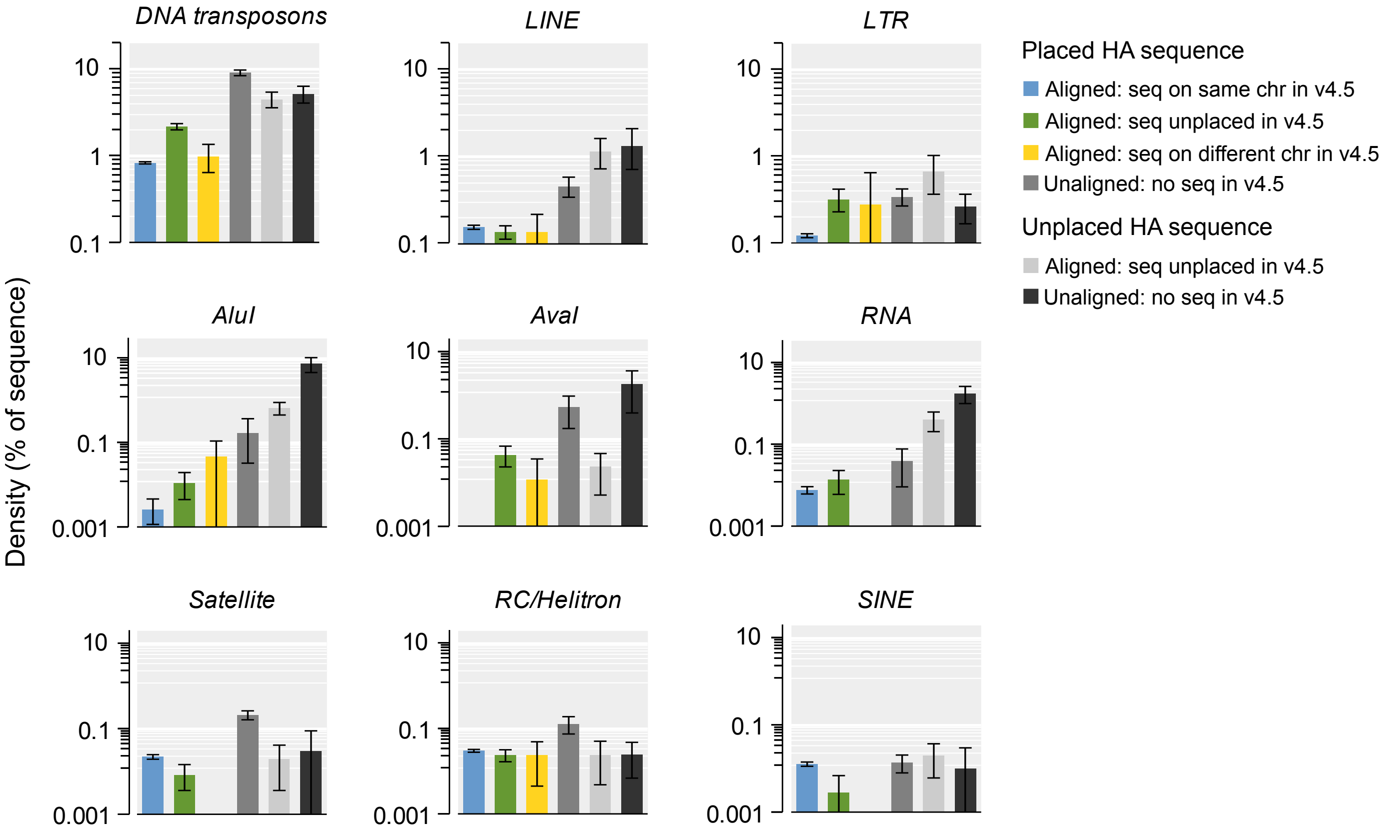

DNA transposons
LINE
LTR
AluI
AvaI
RNA
Satellite
RC/Helitron
SINE
Density (% of sequence)
10
1
0.1
0.001
Placed HA sequence
Aligned: seq on same chr in v4.5
Aligned: seq unplaced in v4.5
Aligned: seq on different chr in v4.5
Unaligned: no seq in v4.5
Unplaced HA sequence
Aligned: seq unplaced in v4.5
Unaligned: no seq in v4.5
